# Discrimination of Four *Cinnamomum* Species with Physico-Functional Properties and Chemometric Techniques: Application of PCA and MDA Models

**DOI:** 10.3390/foods10112871

**Published:** 2021-11-19

**Authors:** Priya Rana, Shu-Yi Liaw, Meng-Shiou Lee, Shyang-Chwen Sheu

**Affiliations:** 1 Department of Tropical Agriculture and International Cooperation, National Pingtung University of Science and Technology, Pingtung 91201, Taiwan; priyarana198933@gmail.com; 2 Department of Business Management, National Pingtung University of Science and Technology, Pingtung 91201, Taiwan; syliaw@mail.npust.edu.tw; 3 Department of Chinese Pharmaceutical Science and Chinese Medicine Resources, China Medical University, Taichung 40402, Taiwan; leemengshiou@mail.cmu.edu.tw; 4 Department of Food Science, National Pingtung University of Science and Technology, Pingtung 91201, Taiwan

**Keywords:** cinnamon, chemometrics, food fraud, identification model, physico-functional

## Abstract

Discrimination of highly valued and non-hepatotoxic *Cinnamomum* species (*C. verum*) from hepatotoxic (*C*. *burmannii*, *C*. *loureiroi*, and *C*. *cassia*) is essential for preventing food adulteration and safety problems. In this study, we developed a new method for the discrimination of four *Cinnamomum* species using physico-functional properties and chemometric techniques. The data were analyzed through principal component analysis (PCA) and multiclass discriminant analysis (MDA). The results showed that the cumulative variability of the first three principal components was 81.70%. The PCA score plot indicated a clear separation of the different *Cinnamomum* species. The training set was used to build the discriminant MDA model. The testing set was verified by this model. The prediction rate of 100% proved that the model was valid and reliable. Therefore, physico-functional properties coupled with chemometric techniques constitute a practical approach for discrimination of *Cinnamomum* species to prevent food fraud.

## 1. Introduction

Cinnamon is one of the important spices and is obtained from the dried inner bark of the evergreen tree belonging to the genus *Cinnamomum*. There are four main economically available species of cinnamon in the spice market, including *C*. *verum* (Ceylon cinnamon, CV), *C*. *burmannii* (Indonesian cinnamon, CB), *C*. *loureiroi* (Vietnamese cinnamon, CL), and *C*. *cassia* (Chinese cinnamon, CC). Consumers’ growing awareness of the health benefits of cinnamon is driving the global cinnamon market, which is expected to reach to US$1.9 billion by 2025 [1]. Because of worldwide demand and the direct relationship between food quality and commercial value, the cinnamon supply chain is susceptible to food fraud. Important types of food fraud are deliberate substitution, dilution or addition, or misrepresentation of food ingredients [2]. According to reports, cinnamon is at very high risk for adulteration involving substitution, and the increased trading of cinnamon substitutes has increased that risk [3].

Many *Cinnamomum* plants are morphologically similar. Some cheaper and hepatotoxic adulterant *Cinnamomum* species, such as *C*. *burmannii*, *C*. *loureiroi*, and *C*. *cassia*, are easily confused with the highly valued and non-hepatotoxic *C*. *verum* [4,5]. Consuming such substitutes, however, is dangerous due to the high amount of coumarin present in comparison to *C*. *verum*. Furthermore, the number of these lower-priced substitutes is increasing in the consumer market [6]. The task of differentiation becomes more challenging and difficult when cinnamon is converted into powder [7]. As a result, distinguishing between cinnamon species is critical for ensuring food quality and avoiding safety issues associated with fraudulent adulteration.

So far, efforts are made for evaluating the quality and safety [8] of cinnamon. Currently, there is increasing demand on governmental agencies and industries to combat the rising threat of food fraud [9]. However, most of the quality-monitoring analytical methods are expensive, have high environmental impact, require skilled analysts, and can only be employed in well-equipped laboratories [10]. To overcome these problems, more recent research trends have emphasized the evaluation of physico-functional properties of food materials, since they could serve as quality control indexes. Physico-functional properties such as pH, moisture content, and density can be determined with limited laboratory resources and are easily accessible in laboratories of less developed or developing countries [11]. Therefore, these analyses can be determined in all steps during routine quality inspections of foods at the industrial or supplier levels.

Chemometric techniques have been successfully employed as useful tools for data analysis in food-related studies [9], for example, assessing food quality, confirming food authenticity, detecting food adulteration, and distinguishing cultivars [11]. At present, there is a growing body of literature discussing the importance of principal component analysis (PCA) and multiclass discriminant analysis (MDA) in discriminating peach varieties, *Boletus edulis* [12], rice varieties [13], and vinegar varieties [14]. Although there has been some discussion of the physical or functional properties of *Cinnamomum* species [15,16], none has reported discrimination of *Cinnamomum* species based on physico-functional properties coupled with chemometric techniques.

This study was aimed at investigating the relative contribution of 13 physico-functional properties of *C. verum*, *C. burmannii*, *C. loureiroi*, and *C. cassia*. PCA was first employed for exploratory purposes and tested the suitability of the physico-functional properties for discrimination of four *Cinnamomum* species. Then, MDA was employed for classification and prediction purposes [17,18].

## 2. Materials and Methods

### 2.1. Sample Collection and Preparation

Twenty cinnamon samples (6 CV, 6 CB, 4 CL, and 4 CC) were collected from different Asian countries over the period 2018 to 2020 (Supplementary Table S1). All samples were identified at the Department of Biological Sciences, National Sun Yat-sen University, Taiwan, based on morpho-anatomical features [19,20]. Dried cinnamon bark samples were crushed manually and then pulverized to powder using a laboratory-scale stainless steel grinder. The powder samples were placed in plastic bags and stored in a vacuum desiccator until use.

### 2.2. Determination of Physico-Functional Properties

A total of 13 physico-functional properties were assessed on each sample.

#### 2.2.1. Bulk Density (BD) and Tapped Density (TD)

The BD and TD were calculated by the ratio of the weight to the unsettled or tapped volume of the sample and expressed in grams per cubic centimeter (g/cm^3^) [21,22].

#### 2.2.2. True Density

The true density was measured by a gas pycnometer (AccuPyc 1340, Micromeritics, Norcross, GA, USA) and calculated using Equation (1) [15].

(1) $$\begin{array}{c} \mathrm{True}\;\mathrm{density}\;\left(\rho_{t}\right)=\left[\frac{W_{s}}{V_{s}}\right]\\ V_{s}=\left[V_{\mathrm{cell}}-\left(\frac{V_{\exp}}{\left(\frac{P_{1}}{P_{2}}\right)-1}\right)\right] \end{array}$$

In Equation (1), W_s_ = weight of the sample (g), V_s_ = volume of the sample (cm^3^), V_cell_ = volume of the cell, V_exp_ = observed volume (experimental), and P_1_ and P_2_ = pressure of the multivolume pycnometer before and after nob revolution, respectively, in psi.

#### 2.2.3. Porosity

Porosity (ε) was determined as the ratio of the difference between true density and bulk density to the true density [15]. The percentage porosity (ε%) was calculated using Equation (2).

(2) $$\;\mathrm{Porosity}\;\left(\varepsilon \%\right)=\left[\left(\;1-\frac{\rho_{b}}{\rho_{t}}\;\right)\times 100\right]$$

where ρ_b_ *=* bulk density (g/cm^3^) and ρ_t_ = true density (g/cm^3^).

#### 2.2.4. pH

Sample pH was determined according to the procedure of Jeong, et al. [23]. One gram of sample was mixed with 40 mL of doubly-deionized (2D) water and shaken for 3 h at 200 rpm. The mixture was then centrifuged (Himac CR 21F, Hitachi Koki Co., Ltd., Tokyo, Japan) at 1294× *g* for 10 min, and the filtrate was collected for pH measurement by a pH meter (sensION ^TM^ + PH3, Hach Lange GmbH, Düsseldorf, Germany). 

#### 2.2.5. Moisture Content

The moisture content of the sample was measured using an automated moisture balance (MA 35, Sartorius Weighing Technology GmbH, Goettingen, Germany) and expressed as % moisture content on a dry basis.

#### 2.2.6. Color

The sample color was determined using a colorimeter (ZE 2000, Nippon Denshoku Industries Co. Ltd., Tokyo, Japan) and evaluated by means of CIELAB coordinates [23]. The total color difference (ΔE) was determined by taking an unsieved sample as a reference and using Equation (3) [11].

(3) $$\Delta E\;={\left[{\Delta L}^{2}+{\Delta a}^{2}+{\Delta b}^{2}\right]}^{\frac{1}{2}}$$

where ΔL = difference in lightness, Δa = difference in red intensity, and Δb = difference in yellow intensity.

#### 2.2.7. Aspect Ratio

The aspect ratio was determined according to the method described by Charles and Alamsjah [11]. Samples were mounted on a microscope slide without overlap of particles and observed under a microscope (Eclipse E100, Nikon Instruments Inc., Melville, NY, USA). The parameters of the aspect ratio, including the particle major axis (l) and minor axis (b), were analyzed by Image-Pro^®^ 10 [24]. The aspect ratio (φ_AR_) was calculated according to Equation (4).

(4) $$\;\mathrm{Aspect}\;\mathrm{ratio}\;\left(\varphi_{\mathrm{AR}}\right)=\left[\frac{\mathrm{minor}\;\mathrm{axis}\;\left(b\right)}{\mathrm{major}\;\mathrm{axis}\;\left(l\right)}\right]$$

#### 2.2.8. Water Absorption Index (WAI) and Water Solubility Index (WSI)

The WAI and WSI were determined by the procedure described by Kraithong, et al. [25]. One gram of sample was added to 10 mL of 2D water and vortexed for 1 min. The suspension was submerged in a water bath at 30 ± 2 °C for 30 min with intermittent stirring and centrifuged at 1294× *g* for 10 min. The supernatant was transferred to a preweighed aluminum moisture dish and dried overnight at 105 °C. The weight of the sediment was recorded. The WAI and WSI were calculated and expressed as g/g of sample and %, respectively, as shown in Equations (5) and (6).

(5) $$\;\mathrm{Water}\;\mathrm{absorption}\;\mathrm{index}\;\left(\mathrm{WAI},\;g/g\right)=\left[\frac{\mathrm{weight}\;\mathrm{of}\;\mathrm{wet}\;\mathrm{sediment}\;\left(g\right)}{\mathrm{dry}\;\mathrm{weight}\;\mathrm{of}\;\mathrm{sample}\;\left(g\right)}\right]$$

(6) $$\mathrm{Water}\;\mathrm{solubility}\;\mathrm{index}\;\left(\mathrm{WSI},\;\%\right)=\left[\left(\;\frac{\mathrm{weight}\;\mathrm{of}\;\mathrm{dried}\;\mathrm{supernatant}\;\left(g\right)}{\mathrm{dry}\;\mathrm{weight}\;\mathrm{of}\;\mathrm{sample}\;\left(g\right)}\;\right)\times 100\right]$$

#### 2.2.9. Oil Absorption Index (OAI)

The OAI was determined as described by Kraithong, et al. [25]. Samples (1 g) were added to commercial soybean oil (10 mL) and centrifuged at 2301× *g* for 20 min. The weight of oil absorbed was recorded. The amount of oil absorbed by the samples was calculated according to Equation (7).

(7) $$\mathrm{Oil}\;\mathrm{absorption}\;\mathrm{index}\;\left(\mathrm{OAI},g/g\right)=\left[\frac{\mathrm{weight}\;\mathrm{of}\;\mathrm{oil}\;\mathrm{absorbed}\;\left(g\right)}{\;\mathrm{weight}\;\mathrm{of}\;\mathrm{sample}\;\left(g\right)}\right]$$

#### 2.2.10. Swelling Power (SP)

The SP was determined according to the method described by Moutaleb, et al. [26]. One gram of sample was mixed with 10 mL of 2D water and then incubated at room temperature for 24 h. The SP was calculated using Equation (8).

(8) $$\mathrm{Swelling}\;\mathrm{power}\;\left(\mathrm{SP},\;\mathrm{mL}/g\right)=\left[\frac{\mathrm{total}\;\mathrm{volume}\;\mathrm{of}\;\mathrm{the}\;\mathrm{swollen}\;\mathrm{sample}\;\left(\mathrm{mL}\right)}{\mathrm{original}\;\mathrm{dry}\;\mathrm{weight}\;\mathrm{of}\;\mathrm{sample}\;\left(g\right)}\right]$$

#### 2.2.11. Emulsifying Activity (EA)

The EA was performed by adapting the method by Chandra, et al. [27]. One gram of sample was mixed with 10 mL of 2D water and 10 mL of soybean oil. The mixture was vortexed thoroughly and centrifuged at 2000× *g* for 5 min. The EA was calculated according to Equation (9).

(9) $$\mathrm{Emulsifying}\;\mathrm{activity}\;\left(\mathrm{EA},\;\%\;\right)=\left[\left(\;\frac{\mathrm{height}\;\mathrm{of}\;\mathrm{emulsified}\;\mathrm{layer}}{\mathrm{total}\;\mathrm{height}\;\mathrm{of}\;\mathrm{mixture}}\;\right)\times 100\right]$$

### 2.3. Data Processing and Analysis

A data matrix consisting of 120 observations (20 cinnamon samples × 6 replicates) and 13 physico-functional variables were used in this study. The replicates were used to enlarge the sample size. One-way ANOVA (analysis of variance) was first performed to determine the significant (*p* < 0.05) variables that could be used to discriminate among *Cinnamomum* species. Then, trials for different combinations of significant variables were conducted for the two selected groups of *Cinnamomum* species using an independent samples *t*-test (*p* < 0.05). Finally, the analysis, providing the best discriminative variables with better discrimination power for the established identification model was used for the chemometric approach.

### 2.4. Chemometric Techniques

IBM SPSS Statistics for Windows, Version 22.0 [28] was employed for chemometric analyses.

#### 2.4.1. Multivariate Analysis of Variance (MANOVA)

Raw data for selected physico-functional properties were subjected to MANOVA to determine the significant interactions between the species and selected variables. Physico-functional properties were taken as the dependent variables, while species were used as the independent variables. Two multivariate tests, Wilk’s lambda (Λ) and Pillai’s trace, were computed to determine significant effects of selected variables on the species.

#### 2.4.2. Principal Component Analysis (PCA)

A total of 120 observations (36 observations each for CV and CB; 24 observations each for CL and CC) were selected. Prior to PCA, we computed the Kaiser–Meyer–Olkin (KMO) measure of sampling adequacy and Bartlett’s test of sphericity to assess the multicollinearity of the data for PCA suitability [29]. A factor extraction method with varimax rotation was employed. The extracted principal components (PCs) with eigenvalues equal to or higher than 1 were used to calculate the PC scores and establish a PCA model. The PC score was calculated according to Zhao, et al. [12], as shown in Equation (10).

(10) $${\mathrm{PC}}_{n}=\left[{\mathrm{FAC}}_{n}\times \sqrt{\lambda_{n}}\right]$$

where PC_n_ = principal component score, FAC_n_ = factor score obtained directly through SPSS analysis, λ = principal component eigenvalue equal to or higher than 1, and n = number of principal component extracted.

#### 2.4.3. Multiclass Discriminant Analysis (MDA)

The PC scores of 120 observations were divided randomly into the training set (83.3%) and the testing set (16.7%) using the Microsoft Excel^®^ 2016 Add-In function, Ablebits tools [30]. The former set included 100 observations of the four species, and the latter set contained the remaining 20 observations. The PC scores of the training set were taken as the input for stepwise discriminant analysis (DA) to build the MDA model [12]. Finally, typical discriminant functions were established for the species distinction models.

## 3. Results and Discussion

### 3.1. Descriptive Statistics of Physico-Functional Properties

The 13 physico-functional properties of *Cinnamomum* species are listed in Table 1, and box-and-whisker plots are shown in Supplementary Figure S1. The bulk density (BD) values for CB (0.45 ± 0.05 g/cm^3^) showed a highly significant difference (*p* < 0.05), while nearly identical values were reported for CV (0.35 ± 0.02 g/cm^3^), CL (0.34 ± 0.01 g/cm^3^), and CC (0.33 ± 0.02 g/cm^3^). Hermanto, et al. [21] reported BD values for CB samples between 0.43 g/cm^3^ and 0.49 g/cm^3^, consistent with our study. A similar trend was followed for the tapped density (TD), where no significant differences (*p* > 0.05) were found for CL (0.58 ± 0.02 g/cm^3^), CC (0.58 ± 0.03 g/cm^3^), and CV (0.57 ± 0.04 g/cm^3^), but that of CB (0.71 ± 0.06 g/cm^3^) was different. Slight variations among the species could be associated with their origin and environmental conditions. The true density varied significantly (*p* < 0.05) among *Cinnamomum* species and ranged from 1.46 ± 0.02 g/cm^3^ (CC) to 1.51 ± 0.00 g/cm^3^ (CV). The increase in the true density of cinnamon samples might be affected by the moisture content [15]. The porosity values were similar for CC (77.40 ± 1.01%), CL (76.71 ± 0.73%), and CV (76.49 ± 1.01%) but not for CB (69.76 ± 3.58%). The higher porosities might be due to drastic changes occurring after the grinding process [31]. The pH values of the four species were reported as moderately acidic pH values ranging between 4.73 ± 0.24 (CV) and 5.04 ± 0.17 (CC). Jeong, et al. [23] reported similar pH values (4.93 to 5.07) for cinnamon powder samples available in the Korean spice market. The observed acidic pH might be associated with the presence of organic compounds (e.g., cinnamaldehyde and cinnamyl acetate) in cinnamon. The highest moisture content was recorded for CC (11.63 ± 0.67%), while the lowest was recorded for CV (9.89 ± 0.44%). These results are in line with the results of Jeong, et al. [23], in which recorded moisture contents ranged from 7.25 to 12.73% in various cinnamon samples. Additionally, the variations in moisture content might have influenced the true densities of the samples [15], which was also evident from our findings. The color differences (ΔEs) showed a significantly (*p* < 0.05) wide range of values from 3.28 ± 0.36 (CC) to 7.89 ± 1.14 (CB). The wide variations observed for ΔE highlighted the diversity of the samples. The aspect ratios varied little between the species, ranging from 1.66 ± 0.27 (CV) to 3.63 ± 0.33 (CB). The similarities in particle aspect ratio might be related to grinding and sieving methods used for sample preparation [11].

On the other hand, similar water absorption indexes (WAIs) were documented for CC (2.93 ± 0.19 g/g) and CL (3.09 ± 0.17 g/g), whereas CV (3.78 ± 0.20 g/g) and CB (5.18 ± 0.82 g/g) showed differences. A high WAI may be associated with large hydrophilic molecules, such as polysaccharides. Other factors, including the nature, concentration and conformation of proteins and the level of protein interaction with water, might also influence the WAI [27]. The water solubility index (WSI) of CL (8.63 ± 0.44%) was high but showed no significant difference (*p* > 0.05) from those of CB (7.89 ± 2.66%) and CC (7.83 ± 1.12%) but differed from that of CV (5.61 ± 1.90%). This trend might be attributable to particle size resulting from similar grinding and sieving processes. The oil absorption index (OAI) values for CV (3.16 ± 0.25 g/g) and CB (2.53 ± 0.20 g/g) differed significantly (*p* < 0.05) from those of CL (2.38 ± 0.09 g/g) and CC (2.32 ± 0.08 g/g). However, OAI is mainly affected by the hydrophilic or hydrophobic nature of the proteins [27], which highlights the partial interdependence between WAI and OAI properties of *Cinnamomum* species. The swelling power (SP) ranged from 3.05 ± 0.19 mL/g (CC) to 8.93 ± 2.02 mL/g (CB). The SP might be affected by the species, particle sizes, and different processing methods or unit operations employed [27]. The emulsifying activity (EA) showed a wide range of values from 1.58 ± 0.39% (CL) to 27.53 ± 13.89% (CB). There are, however, possible explanations, including geographical origin and differences in packaging or storage of cinnamon powder samples [23], which could have affected the physico-functional properties of cinnamon samples. The findings from this study have made several contributions to the current literature by providing useful and practical information on the physico-functional properties of *Cinnamomum* species.

### 3.2. Selection of Discriminative Variables

In general, it is important to understand the major contributing variables (within 13 physico-functional variables) that could provide the maximum information for differentiation of *Cinnamomum* species. We used an independent samples *t*-test to compare the two groups [(CV and CB) ∩ (CL and CC)] and variables to enable the correct identification among different species. The analysis identified the nine most informative physico-functional variables, including BD, true density, porosity, pH, moisture content, color, WAI, WSI, and SP (Supplementary Table S2), with less crossreactivity between the samples.

### 3.3. Multivariate General Linear Analysis

MANOVA was employed to perform multisignificant tests with nine selected physico-functional variables (Supplementary Table S3). The *p*-value was rounded to three decimal places due to generation of very low values, indicating very high significance. A study conducted by Karabagias, et al. [32] supported Pillai’s trace and Wilks’ Λ as the preferred test statistics for MANOVA and suggested the appropriateness of MANOVA by considering possible multi-significant effects of dependent variables on independent variables. In this study, Pillai’s trace (*F* (27,330) = 54.65, *p* = 0.000 < 0.05; Pillai’s trace = 2.45) and Wilks’ Λ (*F* (27,316.06) = 84.52, *p* = 0.000 < 0.05; Wilks’ Λ = 0.00) tests were considered. The results showed the existence of statistically significant multivariate effects of physico-functional properties among the cinnamon samples. Hence, we further applied PCA and MDA for a clear and in-depth understanding of variations among *Cinnamomum* species.

### 3.4. Data Dimensional Reduction through PCA

**Figure 1:**
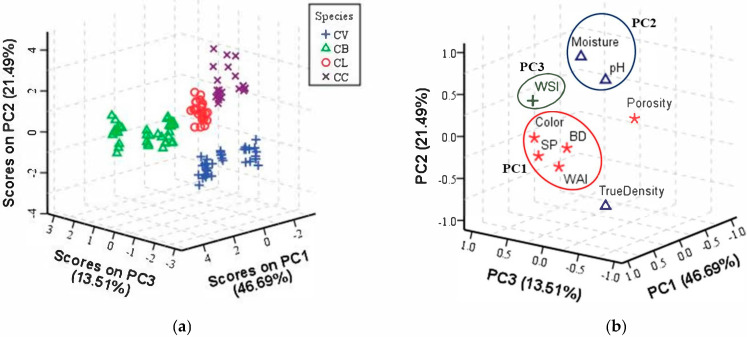
PCA plots of *Cinnamomum* species based on nine physico-functional variables: (**a**) 3-D score plot and (**b**) corresponding loading plot showing positively (with circles) and negatively (without circles) correlated variables. CV: *C*. *verum*; CB: *C*. *burmannii*; CL: *C*. *loureiroi*; CC: *C*. *cassia*; BD: bulk density; WAI: water absorption index; WSI: water solubility index; SP: swelling power.

The dimensionality of the data for nine selected physico-functional variables was reduced to principal components (PCs) using PCA. In the present study, a KMO value of 0.61 and statistically significant (*p <* 0.05) Bartlett’s test of sphericity supported the appropriateness of the data for performing PCA. In addition, the three significant variables (true density, moisture content, and color) determined by one-way ANOVA collectively failed to yield acceptable KMO value (0.45), thus making PCA inapplicable. Therefore, it was not considered in this study. Only the first three PCs presented eigenvalues exceeding 1 (PC1–46.69%, PC2–21.49%, and PC3–13.51%) and explained 81.70% of the cumulative variability (Supplementary Table S4). A three-dimensional (3-D) score plot shows the separation of cinnamon samples into four groups (Figure 1a). The CB samples presented relatively different physico-functional properties and thus formed a distinct group to the left of the score plot. Although CL and CC samples were found close to each other due to similarities in their respective physico-functional properties, significant boundaries were observed between them. Notably, the CV samples were placed towards the bottom of the plot and distinguished from the CB, CL, and CC samples. These results agreed with those from the study by Shawky and Selim [33], which applied PCA to demonstrate a clear separation of CV samples from adulterated cinnamon samples based on near-infrared (NIR) fingerprints. Similarly, Jeong, et al. [23] employed PCA to establish clear variations among different cinnamon powders based on physico-chemical parameters. Our results implied that physico-functional information can be utilized to discriminate among different species of *Cinnamomum* samples.

Moreover, the principal component loading matrix (Supplementary Table S4) of the first three PCs extracted and the corresponding loading plot (Figure 1b) illustrate the relationships among the variables and describe the variables effecting the separation of samples. The CB samples obtained higher scores in PC1 due to high positive loadings for BD, color, WAI, and SP, whilst CV samples showed higher scores in PC1 due to high negative loading for porosity. Similarly, the CC samples reported higher scores in PC2 due to higher values of pH and moisture content, but lower values of true density. Finally, the CL samples projected towards PC3 with a strong positive weight of WSI. This showed the contribution of positively correlated (BD, pH, moisture content, color, WAI, WSI, and SP) and negatively correlated (true density and porosity) variables in explaining variations among the samples.

These findings demonstrated that the cumulative contribution rate of the first three PCs reached 81.70%, indicating that these PCs represented the original variables. From the contained information, the number of original nine selected physico-functional variables was reduced to three new variables called three PCs. Overall, the reliabilities of the three new variables demonstrated that physico-functional analysis with PCA is a promising strategy for discrimination among *Cinnamomum* species. Furthermore, MDA was applied to implement the comprehensive use of *Cinnamomum* physico-functional information from different species to predict the species of a test sample.

### 3.5. Establishment of the MDA Model for Cinnamomum Species

#### 3.5.1. MDA Characteristics

The first three PC scores of 100 observations were used as independent variables, and *Cinnamomum* species were used as grouping variables. The highlighted MDA characteristics of nine physico-functional variables using stepwise DA are summarized (Supplementary Table S5). The results showed that three significant discriminant functions (DF1 = 60.30%, DF2 = 39.20%, and DF3 = 0.50%) accounted for 100% of the total variance. We exclude the discussion of DF3 since it represented a very small fraction of the total information. The Wilks’ Λ values for DF1 (χ^2^ = 443.66, *p* = 0.000 < 0.05) and DF2 (χ^2^ = 207.26, *p* = 0.000 < 0.05) were 0.10 and 0.11, respectively. The existence of small Wilks’ Λ and large chi-square (χ^2^) values indicated significantly high discriminatory ability of a function and that the groups appeared to differ [11]. Moreover, DF1 exhibited a high eigenvalue (10.89) and canonical correlation of 0.96, followed by DF2 with an eigenvalue of 7.08 and canonical correlation of 0.94. These findings also revealed that a larger eigenvalue explained more variance in the grouping variable in the function test. Similarly, a higher canonical correlation indicated significant differences in physico-functional properties among *Cinnamomum* species.

#### 3.5.2. Identification Model for Cinnamon Samples

The identification model was developed using stepwise DA to correctly identify cinnamon samples based on nine physico-functional variables. Fisher’s linear discrimination functions were established for the species distinction models according to the following Equations (11)–(14):

(11) $$\;\mathrm{CV}:{\;Y}_{1}\left(x\right)=-10.97-\;3.22x_{1}-8.09x_{2}-4.97x_{3}$$

(12) $$\;\mathrm{CB}:\;Y_{2}\left(x\right)=-12.71+7.40x_{1}+2.36x_{2}+3.55x_{3}$$

(13) $$\;\mathrm{CL}:\;Y_{3}\left(x\right)=-3.82-2.01x_{1}+2.87x_{2}+1.21x_{3}$$

(14) $$\;\mathrm{CC}:\;Y_{4}\left(x\right)=-10.81-\;4.31x_{1}+6.18x_{2}+1.13x_{3}$$

where Y_1_(*x*), Y_2_(*x*), Y_3_(*x*), and Y_4_(*x*) are the identification values for CV, CB, CL, and CC, respectively. *x*_1_, *x*_2_, and *x*_3_ are the values of the first three PC scores.

The values of the first three PC scores of 100 training observations were taken into the established identification functions to validate the functions. Out of 100 observations, two observations originating from CB and one observation originating from CC were misclassified as CL. This could be explained by a minor and unavoidable experimental handling error or the close relationship among CB, CL, and CC. As shown in Table 2, the correct identification rates were 100%, 93.30%, 100%, and 95% for CV, CB, CL, and CC, respectively. The overall correct rate of 97% showed that the established identification model was feasible. Therefore, MDA could be employed to build a distinction model for *Cinnamomum* species with a high percentage of correct identification based on physico-functional properties. In order to establish a full-scale quality evaluation and discrimination system for *Cinnamomum* species, collecting more cinnamon samples from different species should be required in the further study.

**Figure 2:**
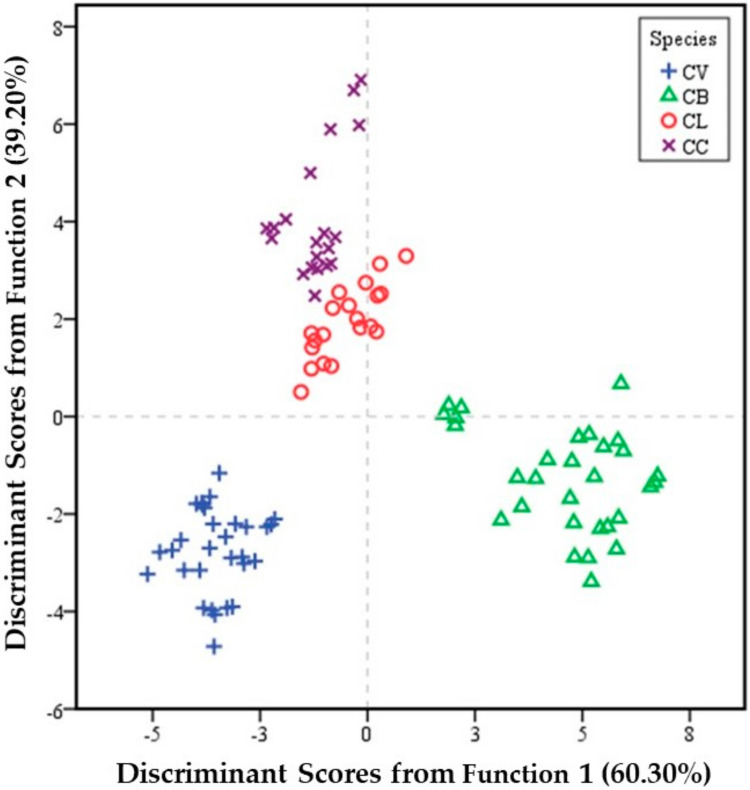
2-D score plot of *Cinnamomum* species based on the two discriminant functions. CV: *C. verum*; CB: *C. burmannii*; CL: *C. loureiroi*; CC: *C. cassia*.

A two-dimensional (2-D) score plot (DF1 × DF2) represents the qualitative identification of *Cinnamomum* species (Figure 2). The results showed that CL and CC samples were spread out in the second quadrant, while CV samples were located in the third quadrant. On the other hand, CB samples were distributed in the fourth quadrant. Therefore, CV samples were completely separated from other samples. We concluded that there was a good cluster result for *Cinnamomum* species based on the first two DFs.

#### 3.5.3. Analytical Model Prediction for Cinnamon Samples

To further test the reliability of the established identification model, the 20 testing observations were set into the four identification functions. The function with a larger value determined the predicted species (Supplementary Table S6). The results of the testing set to validate the built model showed a 100% prediction rate for the assigned samples to their respective categories (Table 2). Therefore, the established identification model was valid and reliable.

## 4. Conclusions

This study presented a valid and reliable model for *Cinnamomum* discrimination with the potential use of selected physico-functional variables coupled with chemometric techniques. By combining the PCA and MDA techniques, a relationship was established between the physico-functional properties and the *Cinnamomum* species. Additionally, by applying PCA, the training and testing of the MDA model have become feasible. The data correct identification and prediction rates realized by using the MDA model provide a checkpoint for food authorities. The combination of characteristic physico-functional variables with different *Cinnamomum* species constitutes the novelty of the present work designed to ensure future food safety. However, this method may be time-consuming way to train and test the model. Future research is recommended on the integration of feature selection and data mining approaches to decrease training time and accelerate learning from testing a large number of samples.

## Figures and Tables

**Table 1:** Descriptive statistics for physico-functional properties of cinnamon samples from four *Cinnamomum* species.

Variables	*Cinnamomum* Species															
	*C*. *verum* (*n* = 6)				*C*. *burmannii* (*n* = 6)				*C*. *loureiroi* (*n* = 4)				*C*. *cassia* (*n* = 4)			
	Mean	SD	Min	Max	Mean	SD	Min	Max	Mean	SD	Min	Max	Mean	SD	Min	Max
Bulk density (g/cm^3^)	0.35 ^b^	0.02	0.33	0.39	0.45 ^a^	0.05	0.39	0.53	0.34 ^b c^	0.01	0.32	0.36	0.33 ^c^	0.02	0.31	0.36
Tapped density (g/cm^3^)	0.57 ^b^	0.04	0.50	0.65	0.71 ^a^	0.06	0.63	0.82	0.58 ^b^	0.02	0.54	0.63	0.58 ^b^	0.03	0.54	0.63
True density (g/cm^3^)	1.51 ^a^	0.00	1.50	1.52	1.49 ^b^	0.01	1.48	1.50	1.48 ^c^	0.01	1.47	1.49	1.46 ^d^	0.02	1.42	1.48
Porosity (%)	76.49 ^a^	1.01	74.01	78.36	69.76 ^b^	3.58	64.44	73.69	76.71 ^a^	0.73	75.55	78.39	77.40 ^a^	1.01	75.72	79.24
pH	4.73 ^c^	0.24	4.42	5.11	4.90 ^b^	0.10	4.78	5.08	4.93 ^b^	0.09	4.83	5.08	5.04 ^a^	0.17	4.75	5.21
Moisture content (%)	9.89 ^d^	0.44	9.03	10.58	11.15 ^b^	0.40	10.20	11.85	10.80 ^c^	0.50	10.03	11.76	11.63 ^a^	0.67	10.57	12.92
Color	3.85 ^c^	0.78	2.94	5.22	7.89 ^a^	1.14	5.90	9.36	5.76 ^b^	0.58	5.06	6.66	3.28 ^d^	0.36	2.81	3.79
Aspect ratio	1.66 ^c^	0.27	1.11	2.17	3.63 ^a^	0.33	3.26	4.67	2.85 ^b^	0.14	2.44	3.09	2.78 ^b^	0.04	2.69	2.85
Water absorption index (g/g)	3.78 ^b^	0.20	3.27	4.07	5.18 ^a^	0.82	4.17	6.40	3.09 ^c^	0.17	2.90	3.33	2.93 ^c^	0.19	2.58	3.15
Water solubility index (%)	5.61 ^b^	1.90	2.81	8.62	7.89 ^a^	2.66	4.14	12.68	8.63 ^a^	0.44	7.95	9.42	7.83 ^a^	1.12	6.11	9.29
Oil absorption index (g/g)	3.16 ^a^	0.25	2.64	3.48	2.53 ^b^	0.20	2.30	2.81	2.38 ^c^	0.09	2.24	2.51	2.32 ^c^	0.08	2.21	2.44
Swelling power (mL/g)	4.56 ^b^	0.17	4.19	4.80	8.93 ^a^	2.02	5.20	11.40	3.53 ^c^	0.25	3.10	3.90	3.05 ^c^	0.19	2.70	3.40
Emulsifying activity (%)	2.97 ^b^	0.48	2.27	4.84	27.53 ^a^	13.89	7.14	46.34	1.58 ^b^	0.39	0.82	2.61	1.72 ^b^	0.32	1.40	2.54

Data is mean of six replicates. Mean values followed by different superscripts (a–d) within the same row are significantly different (*p* < 0.05) based on Duncan’s test (One-way ANOVA). *n* is the number of samples and SD is standard deviation.

**Table 2:** Correct identification and prediction rates of the training and testing sets based on the MDA model.

	**Actual Species**	**Species Discriminated by Model**				**Total**	**Correct Identification** **Rate (%)**
		**CV**	**CB**	**CL**	**CC**		
Training set	CV	30	0	0	0	30	100
	CB	0	28	2	0	30	93.30
	CL	0	0	20	0	20	100
	CC	0	0	1	19	20	95
	Total	30	28	23	19	100	97
	**Actual Species**	**Species Discriminated by Model**				**Total**	**Correct Prediction** **Rate (%)**
		**CV**	**CB**	**CL**	**CC**		
Testing set	CV	6	0	0	0	6	100
	CB	0	6	0	0	6	100
	CL	0	0	4	0	4	100
	CC	0	0	0	4	4	100
	Total	6	6	4	4	20	100

CV: *C. verum*; CB: *C. burmannii*; CL: *C. loureiroi*; CC: *C. cassia*.

## Data Availability

The data that support the findings of this study are available from the corresponding author upon reasonable request.

## Supplementary Materials

The following are available online at https://www.mdpi.com/article/10.3390/foods10112871/s1, Figure S1: Box-and-whisker plots representing different physico-functional properties for the studied *Cinnamomum* species, Table S1: Detailed information on the *Cinnamomum* samples used in the study, Table S2: Independent samples *t*-test results of the nine selected discriminative physico-functional variables, Table S3: MANOVA results of *Cinnamomum* species using nine physico-functional variables, Table S4: Total variance explained and principal component loading matrix, Table S5: Stepwise DA characteristics of *Cinnamomum* species using nine physico-functional variables, Table S6: Prediction result of the testing set by the MDA model.
