# Supplementary material for: Discrimination of Four Cinnamomum Species with Physico-Functional Properties and Chemometric Techniques: Application of PCA and MDA Models
Source: Foods. 2021 Nov 19;10(11):2871. doi: 10.3390/foods10112871 (PMC8619511; doi:10.3390/foods10112871)
Supplement: Supplementary file 1 [file foods-10-02871-s001.zip › Supplementary figure.pdf]

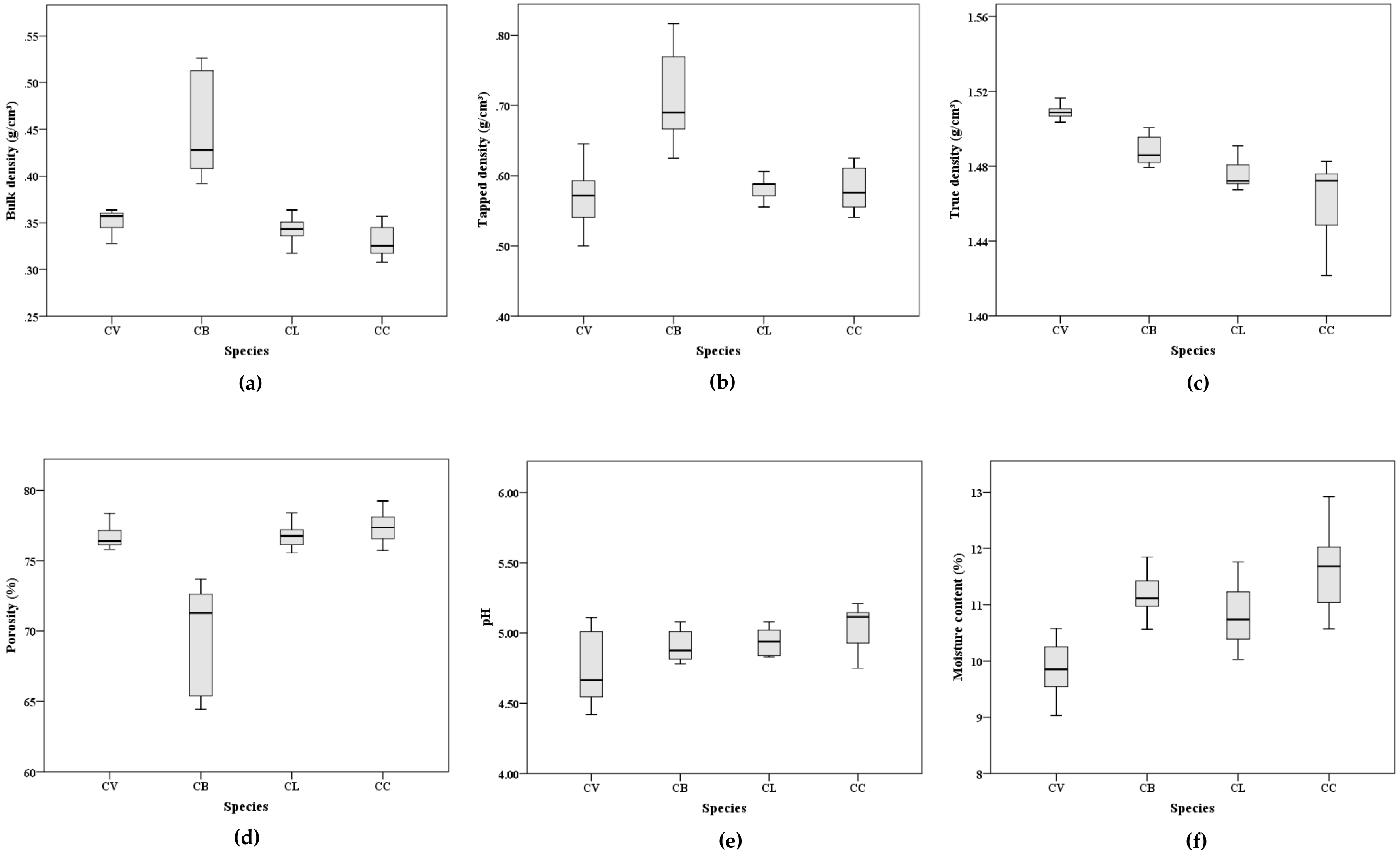
Bulk density (g/cm³)
.55
.50
.45
.40
.35
.30
.25
CV
CB
CL
CC
Species
(a)
Tapped density (g/cm³)
.80
.70
.60
.50
.40
CV
CB
CL
CC
Species
(b)
True density (g/cm³)
1.56
1.52
1.48
1.44
1.40
CV
CB
CL
CC
Species
(c)
Porosity (%)
80
75
70
65
60
CV
CB
CL
CC
Species
(d)
pH
6.00
5.50
5.00
4.50
4.00
CV
CB
CL
CC
Species
(e)
Moisture content (%)
13
12
11
10
9
8
CV
CB
CL
CC
Species
(f)

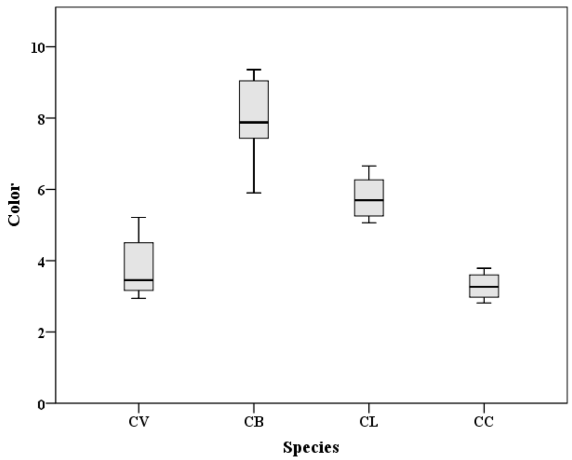

Color
Species
CV
CB
CL
CC


**(g)**

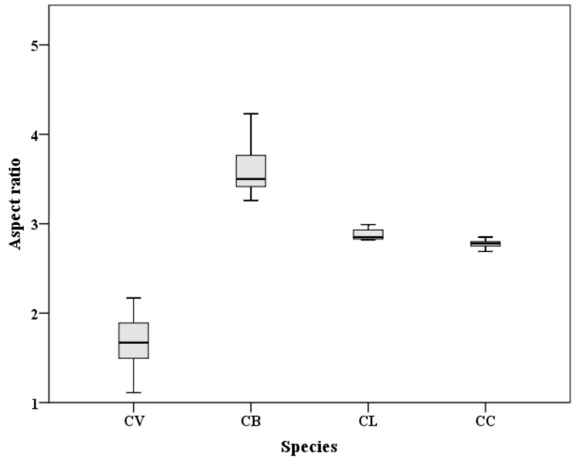

Aspect ratio
Species
CV
CB
CL
CC


**(h)**

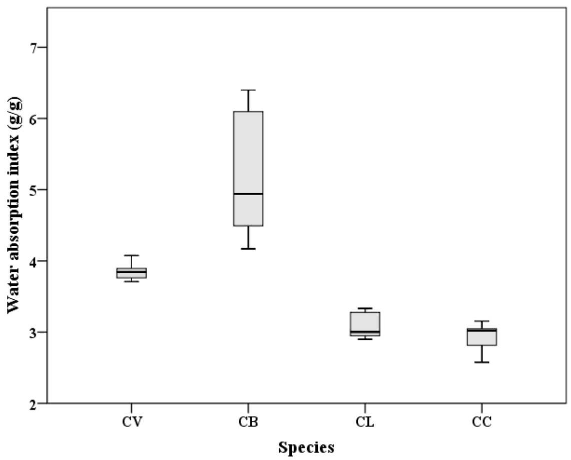

Water absorption index (g/g)
Species
CV
CB
CL
CC


**(i)**

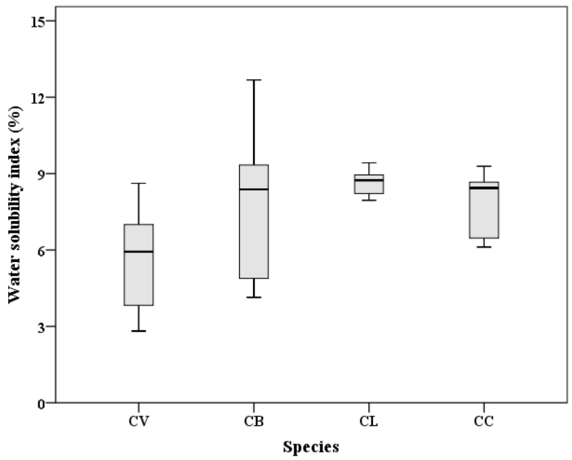

Water solubility index (%)
Species
CV
CB
CL
CC


**(j)**

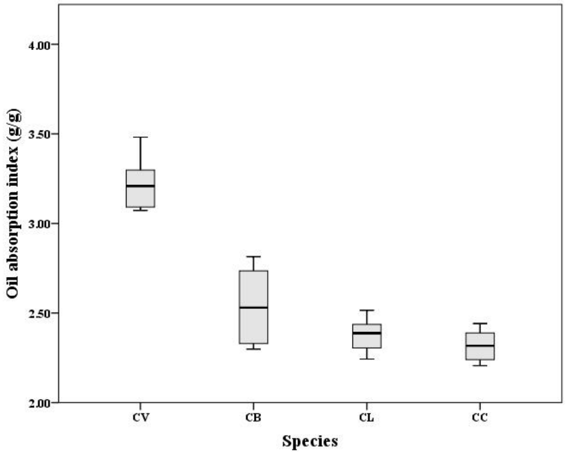

Oil absorption index (g/g)
Species
CV
CB
CL
CC


**(k)**

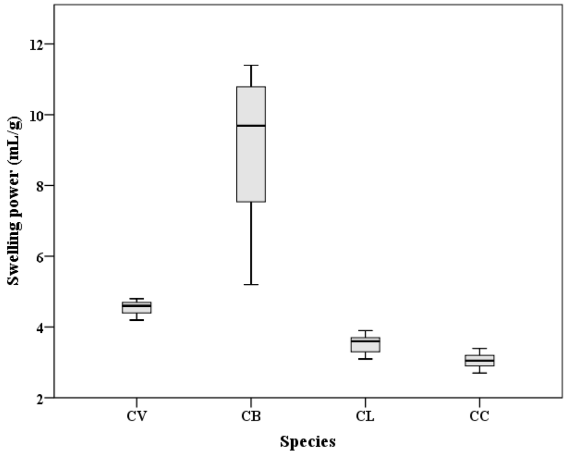

Swelling power (mL/g)
Species
CV
CB
CL
CC


**(l)**

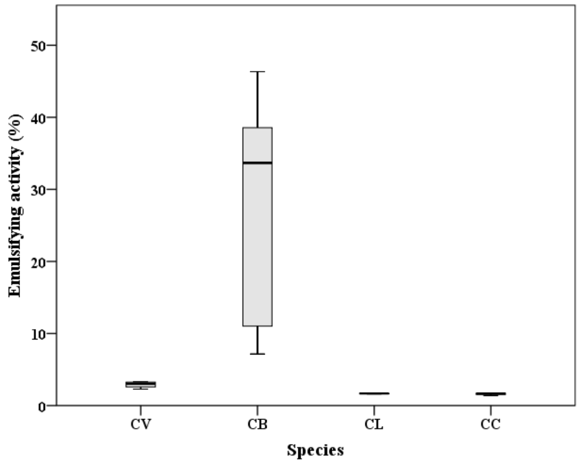


**(m)**

**Figure S1**. Box-and-whisker plots representing different physico-functional properties for the studied *Cinnamomum* species: **(a)** bulk density, **(b)** tapped density, **(c)** true density, **(d)** porosity, **(e)** pH, **(f)** moisture content, **(g)** color, **(h)** aspect ratio, **(i)** water absorption index, **(j)** water solubility index, **(k)** oil absorption index, **(l)** swelling power, and **(m)** emulsifying activity. CV: *C. verum*, CB: *C. burmannii*, CL: *C. loureiroi*, and CC: *C. cassia*.
