# Supplementary material for: Discrimination of Four Cinnamomum Species with Physico-Functional Properties and Chemometric Techniques: Application of PCA and MDA Models
Source: Foods. 2021 Nov 19;10(11):2871. doi: 10.3390/foods10112871 (PMC8619511; doi:10.3390/foods10112871)
Supplement: Supplementary file 1 [file foods-10-02871-s001.zip › Supplementary tables.pdf]

Table S1. Detailed information on the *Cinnamomum* samples used in the study.

Sample no.	Species	Collected location	Collected year
1	*C. verum*	Uswetakeyyawa Wattala, Sri Lanka	2018
2		Dambulla, Sri Lanka	2020
3		Dambulla, Sri Lanka	2020
4		Kerala, India	2018
5		Kerala, India	2018
6		Kerala, India	2018
7	*C. burmannii*	East Java, Indonesia	2019
8		East Java, Indonesia	2019
9		East Java, Indonesia	2018
10		East Java, Indonesia	2018
11		South Jakarta, Indonesia	2019
12		South Jakarta, Indonesia	2019
13	*C. loureiroi*	Vietnam	2019
14		Vietnam	2019
15		Ho Chi Minh City, Vietnam	2018
16		Ho Chi Minh City, Vietnam	2020
17	*C. cassia*	Tainan, Taiwan	2018
18		Tainan, Taiwan	2018
19		Tainan, Taiwan	2019
20		Tainan, Taiwan	2019

Table S2. Independent samples *t*-test results of the nine selected discriminative physico-functional variables.

Variables	*Cinnamomum* species											
	Group 1						*Group 2*					
	C. verum		*C. burmannii*		***t*-test results**		*C. loureiroi*		*C. cassia*		***t*-test results**	
	Mean	SD	Mean	SD	*t*	*p*	Mean	SD	Mean	SD	*t*	*p*
**BD**	0.35	0.02	0.45	0.05	−10.557	0.000	0.34	0.01	0.33	0.02	3.376	0.002
**True density**	1.51	0.00	1.49	0.01	15.704	0.000	1.48	0.01	1.46	0.02	2.746	0.010
**Porosity**	76.49	1.01	69.76	3.58	10.845	0.000	76.71	0.73	77.40	1.01	−2.746	0.009
**pH**	4.73	0.24	4.90	0.10	−3.914	0.000	4.93	0.09	5.04	0.17	−2.731	0.010
**Moisture content**	9.89	0.44	11.15	0.40	−12.719	0.000	10.80	0.50	11.63	0.67	−4.870	0.000
**Color**	3.85	0.78	7.89	1.14	−17.496	0.000	5.76	0.58	3.28	0.36	17.873	0.000
**WAI**	3.78	0.20	5.18	0.82	−10.002	0.000	3.09	0.17	2.93	0.19	2.873	0.006
**WSI**	5.61	1.90	7.89	2.66	−4.184	0.000	8.63	0.44	7.83	1.12	3.257	0.003
**SP**	4.56	0.17	8.93	2.02	−12.908	0.000	3.53	0.25	3.05	0.19	7.465	0.000

The significant ($p < 0.05$) physico-functional variables were selected based on selected two groups [(CV and CB) ∩ (CL and CC)].
BD: bulk density; WAI: water absorption index; WSI: water solubility index; SP: swelling power.

Table S3. MANOVA results of *Cinnamomum* species using nine physico-functional variables.

**Test**	**Value**	***F*- value**	**Hypothesis df**	**Error df**	***p*-value**
Pillai's trace	2.45	54.65	27	330	0.000
Wilks' Λ	0.00	84.52	27	316.06	0.000
Hotelling's trace	27.45	108.44	27	320	0.000
Roy's largest root	15.72	192.12	9	110	0.000

Wilks' Λ: Wilks' lambda

Table S4. Total variance explained and principal component loading matrix.

	Principal components		
	PC1	PC2	PC3
Total Variance explained			
Eigenvalue	4.20	1.93	1.22
% Variance	46.69	21.49	13.51
Cumulative %	46.69	68.18	81.70
Variable loading			
BD	**0.94**	0.06	−0.21
True density	0.18	**−0.82**	−0.20
Porosity	**−0.93**	−0.13	0.19
pH	−0.01	**0.61**	−0.07
Moisture content	0.15	**0.88**	0.17
Color	**0.88**	0.08	0.29
WAI	**0.91**	−0.20	−0.08
WSI	−0.01	0.61	**0.96**
SP	**0.90**	−0.13	0.22

The high loadings are in bold.
BD: bulk density; WAI: water absorption index; WSI: water solubility index; SP: swelling power.

Table S5. Stepwise DA characteristics of *Cinnamomum* species using nine physico-functional variables.

**DF**	**Eigenvalue**	**% Variance**	**Cumulative %**	**Canonical correlation**	**Wilks' Λ**	**χ^2 value**	**df**	***p*-value**
1	10.89	60.30	60.30	0.96	0.10	443.66	9	0.000
2	7.08	39.20	99.50	0.94	0.11	207.26	4	0.000
3	0.09	0.50	100.00	0.28	0.92	7.78	1	0.005

DF: discriminant function; df: degree of freedom; Wilks' Λ: Wilks' lambda; χ^2: Chi-square.

Table S6. Prediction result of the testing set by the MDA model.

Sample	Actual species	Values of linear discriminant functions				Predicted species
		CV	CB	CL	CC	
1	CV	**9.88**	−22.41	−9.26	−23.18	CV
2	CV	**8.29**	−22.74	−8.54	−20.89	CV
3	CV	**11.33**	−24.77	−8.96	−22.12	CV
4	CV	**12.34**	−29.65	−8.06	−17.08	CV
5	CV	**7.54**	−24.28	−7.74	−18.13	CV
6	CV	**9.60**	−29.81	−6.74	−13.86	CV
7	CB	−21.49	**15.58**	−11.76	−29.84	CB
8	CB	−32.68	**15.20**	−5.71	−17.79	CB
9	CB	−19.27	**−2.51**	−4.44	−12.85	CB
10	CB	−17.57	**5.45**	−9.08	−23.10	CB
11	CB	−21.46	**15.22**	−13.08	−27.98	CB
12	CB	−17.99	**8.68**	−11.42	−25.03	CB
13	CL	−7.67	−19.76	**−1.83**	−7.15	CL
14	CL	−13.39	−17.11	**−0.15**	−3.93	CL
15	CL	−8.99	−17.42	**−2.15**	−8.36	CL
16	CL	−12.56	−18.45	**0.04**	−3.36	CL
17	CC	−22.11	−20.02	5.42	**8.65**	CC
18	CC	−17.02	−21.57	3.51	**4.96**	CC
19	CC	−15.31	−26.46	4.71	**8.59**	CC
20	CC	−33.88	−18.32	10.68	**19.20**	CC

The functions with larger values are in bold.
